# Highly Plasticized Lanthanide Luminescence for Information Storage and Encryption Applications

**DOI:** 10.1002/advs.202105108

**Published:** 2022-01-12

**Authors:** Yawei Liu, Kelu Zhao, Yubin Ren, Sikang Wan, Chenjing Yang, Jingjing Li, Fan Wang, Chunying Chen, Juanjuan Su, Dong Chen, Yuliang Zhao, Kai Liu, Hongjie Zhang

**Affiliations:** ^1^ College of Materials Science and Opto‐Electronic Technology University of Chinese Academy of Sciences Beijing 100049 China; ^2^ State Key Laboratory of Rare Earth Resource Utilization Changchun Institute of Applied Chemistry Chinese Academy of Sciences Changchun 130022 China; ^3^ Key Laboratory of Organic Optoelectronics & Molecular Engineering of Ministry of Education Department of Chemistry Tsinghua University Beijing 100084 China; ^4^ College of Energy Engineering and State Key Laboratory of Fluid Power and Mechatronic Systems Zhejiang University Hangzhou 310027 China; ^5^ National Center for Nanoscience and Technology of China Beijing 100190 China

**Keywords:** encryption, information storage, lanthanide organogel, luminescence, plasticity

## Abstract

The development of new storage media to meet the demands for diverse information storage scenarios is a great challenge. Here, a series of lanthanide‐based luminescent organogels with ultrastrong mechanical performance and outstanding plasticity are developed for patterned information storage and encryption applications. The organogels possessing outstanding mechanical properties and tunable luminescent colors are prepared by electrostatic and coordinative interactions between natural DNA, synthetic ligands, and rare earth (RE) ions. The organogel‐REs can be stretched by 180 times and show an ultrastrong breaking strength of 80 MPa. A series of applications with both information storage and encryption, such as self‐information pattern, quick response (QR) code, and barcode, are successfully demonstrated by the organogel‐REs. The developed information storage systems have various advantages of good processability, high stretchability, excellent stability, and versatile design of information patterns. Therefore, the organogel‐RE‐based information storage systems are suitable for applications under different scenarios, such as flexible devices under repeating rude operations. The advancements will enable the design and development of luminescent organogel‐REs as information storage and encryption media for various scenarios.

## Graphical abstract

Highly plasticized and mechanically ultrastrong lanthanide organogels are fabricated by supramolecular complexation. Patterned information storage and encryption applications are realized in a versatile way by the flexible luminescence of organogels. This work offers new storage media to meet the demands for diverse information storage scenarios.

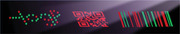

## 1 Introduction

With the explosive growth of big data and the increasing importance of information security, the demand for developing new types of storage media for information storage and encryption has increased dramatically.^[^ ^1^, ^2^, ^3^, ^4^, ^5^, ^6^, ^7^, ^8^ ^]^ Lanthanide‐based materials with distinct optical performances,^[^ ^9^, ^10^, ^11^, ^12^, ^13^, ^14^, ^15^ ^]^ such as multiple excited wavelengths,^[^ ^16^ ^]^ tunable color emission,^[^ ^17^ ^]^ different luminescence lifetime,^[^ ^18^ ^]^ and good luminescent stability,^[^ ^19^, ^20^ ^]^ have displayed a great value in information storage and anticounterfeiting applications.^[^ ^21^ ^]^ Common strategies developed to store information strongly rely on the preparation of luminescent inks based on lanthanide‐doped nanomaterials.^[^ ^22^, ^23^, ^24^, ^25^, ^26^ ^]^ Information patterns are generally prepared by printing luminescent inks on external supporters.^[^ ^22^, ^27^, ^28^ ^]^ However, due to the weak mechanical strength, low toughness, fragility, or poor integrity of the inks,^[^ ^29^, ^30^ ^]^ the stored information may easily be damaged under deformations and thus the systems could not be applied for information storage in complex scenarios, such as flexible devices. Innovations of novel lanthanide‐based luminescent materials with high mechanical strength, excellent processability, and great integrity are required to meet the demands for information storage under different circumstances.^[^ ^31^ ^]^

In addition to the need for luminescent materials with good mechanical properties,^[^ ^32^ ^]^ the developments of encoding and decoding systems are also important for information storage, especially for information encryption.^[^ ^33^, ^34^ ^]^ Previous information storage systems prepared using functional materials are generally limited to simple patterns, making it hard to design information encryption and thus greatly limiting their widespread applications.^[^ ^21^, ^35^, ^36^ ^]^ The advancements of image processing techniques will enable the design of complex patterns for information encoding and decoding, which could greatly broaden the designs of information storage styles. In addition, information encryption could be implemented in the luminescence of the complex patterns. For example, information could simultaneously be stored in the apparent patterns and encrypted in the luminescent color, intensity, or lifetime. Therefore, the developments of novel materials and encoding/decoding techniques will provide an ample space for the designs of information storage systems and intrigue the emergences of diverse information storage styles for multiscenario applications.

In this work, lanthanide‐based luminescent organogel‐REs are developed through noncovalent interactions between natural DNA, synthetic ligands and rare earth (RE) ions. The organogel‐REs possess very good mechanical properties and show an ultrahigh breaking strength of 80 MPa, which surpasses most gel materials.^[^ ^37^, ^38^, ^39^, ^40^, ^41^ ^]^ By utilizing the luminescent colors of the organogel‐REs as the binary codes of “0” and “1,” information storage and encryption are realized in complex patterns of organogel‐REs, such as self‐information pattern, quick‐response (QR) code and barcode. Information encoded in the apparent patterns could easily be retrieved using a standard decoding program, while information encrypted in the pattern colors could only be deciphered from the color image under 365 nm illumination using a color recognition and shape detection program. The developed information storage systems possess excellent stability and could maintain their integrity even under repeated stretching, bending and twisting actions. The results suggest that lanthanide‐based luminescent organogel‐REs could be adapted for information storage and encryption in various complex scenarios, paving the way for their practical applications.

## 2 Results and Discussions

**Figure 1:**
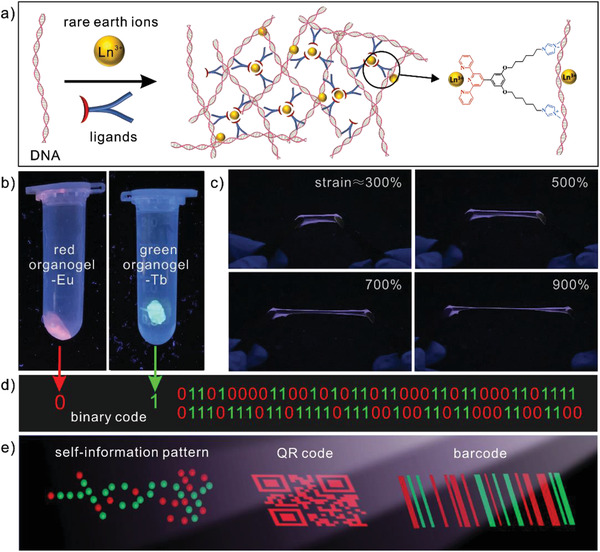
Design of lanthanide‐based luminescent organogels for information storage and encryption. a) Schematics showing the preparation of luminescent organogels via electrostatic and coordinative interactions between DNA, TPBI ligands, and rare earth ions. Each TPBI ligand could electrostatically bind to two DNA chains and each RE ion could coordinate with three TPBI ligands, both of which could perform as crosslinkers for the organogel matrix. b) Red and green luminescence of organogel‐Eu (left) and organogel‐Tb (right) under 365 nm illumination, respectively. c) Organogel‐Eu showing red luminescence at large strains. d) Applications of luminescent organogel‐REs for information storage. Red and green luminescent colors represent the binary codes of “0” and “1,” respectively. e) Designs of self‐information pattern, QR code, and barcode based on luminescent organogel‐REs for information storage and encryption.

Luminescent organogel‐REs are prepared by electrostatic and coordinative interactions between natural DNA, synthetic ligands, and RE ions, as shown in **Figure** **1**a. Natural DNA is double‐strand salmon sperm DNA with negative charges. Synthetic ligands, 1,1′‐(((5‐([2,2′:6′,2″‐terpyridin]‐4′‐yl)‐1,3‐henylene)bis(oxy))bis(hexane‐6,1‐diyl))bis(3‐ethyl‐*H*‐imidazol‐3‐ium) (TPBI), are prepared via four steps, as shown in the scheme of Figure S1 in the Supporting Information; the detailed ^1^H and ^13^C NMR spectra of intermediate products and TPBI are shown in Figures S2–S9 in the Supporting Information. Synthetic ligands are designed with two positively charged end groups, which could electrostatically interact with negatively charged DNA, and one terpyridine section, which could coordinate with RE ions. RE ions are trivalent lanthanide ions, including La^3+^, Ce^3+^, Sm^3+^, Eu^3+^, Tb^3+^, and Dy^3+^. After mixing DNA, TPBI ligands, and RE ions in an aqueous solution, each TPBI ligand with two positively charged end groups could electrostatically bind with two negatively charged DNA and each RE ion could coordinate with three TPBI ligands via their terpyridine sections. The successful coordination between RE ions and TPBI ligands is supported by the fluorescent color of RE ions and the successful electrostatic interaction between DNA and TPBI ligands is supported by the formation of DNA organogel in the absence of RE ions, as shown in Figure S10 in the Supporting Information. Therefore, both of TPBI ligands and RE ions could perform as noncovalent crosslinkers and lead to the formation of a 3D crosslinked matrix. After centrifugation, lyophilization and swelling in dimethyl sulfoxide (DMSO), luminescent organogel‐REs are obtained. The content of DMSO in the organogels measured by thermogravimetry is roughly 60%, as shown in Figures S11 and S12 in the Supporting Information.

The luminescence of organogel‐REs is attributed to the coordination of RE ions with TPBI ligands and the luminescent color strongly depends on the type of RE ions. Red and green luminescence is thus achieved under 365 nm illumination in organogel‐Eu and organogel‐Tb, respectively, as shown in Figure 1b. The luminescent intensity of organogel‐REs increases as the concentration of RE ions increases, as shown in Figure S13 in the Supporting Information. The organogel‐REs possess good luminescent stability and still show excellent luminescence after 10 months. Since organogel‐REs are formed via noncovalent intermolecular interactions, such as electrostatic complexation and coordination, organogel‐REs display excellent processability, flexibility, stretchability, and stability. Organogel‐REs could maintain their luminescence even when stretched up to 900%, as shown in Figure 1c. Because red and green luminescent colors could represent the binary codes of “0” and “1,” respectively, organogel‐REs are suitable for information storage, as shown in Figure 1d. Different strategies, such as self‐information pattern, QR code, and barcode, which take full advantages of the unique properties of organogel‐REs, are developed for information storage and encryption, as shown in Figure 1e.

**Figure 2:**
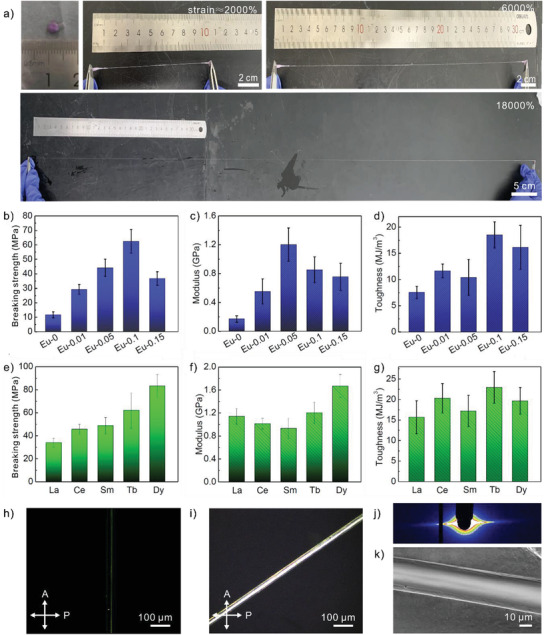
Mechanical performance of the luminescent organogel‐REs. a) The stretchable organogel‐Eu‐0.05. The organogel‐Eu‐0.05 can be stretched by about 20, 60, and 180 times, respectively. b) Breaking strength, c) modulus, and d) toughness of the organogel‐Eu fibers containing different amounts of Eu. The concentration of TPBI ligands is fixed at 5 mg mL^−1^. Eu‐0, Eu‐0.01, Eu‐0.05, Eu‐0.1, and Eu‐0.15 denote the mole ratio of Eu to ligand = 0:3, 0.01:3, 0.05:3, 0.1:3, and 0.15:3, respectively. e) Breaking strength, f) modulus, and g) toughness of organogel‐La, organogel‐Ce, organogel‐Sm, organogel‐Tb, and organogel‐Dy fibers. The mole ratio of RE to ligand is fixed at 0.1:3. h,i) Optical images of an organogel‐Dy fiber under crossed polarizer and analyzer at different angles. The fiber appears dark at ≈0° and becomes bright at ≈45°, suggesting that DNA strands are orientationally ordered along the fiber. j) 2D SAXS of an organogel‐Dy fiber, showing a strong angular dependence. k) SEM image of an organogel‐Dy fiber.

Organogel‐REs possess great ductility and could be elongated to 2000%, 6000%, and 18 000% of their original length, as shown in **Figure** **2**a. Because of their excellent ductility, organogel‐REs could easily be stretched into organogel‐RE fibers, even when the strain is removed. To systematically characterize the mechanical properties of organogel‐REs, the mechanical performances of organogel‐RE fibers prepared using different concentrations and types of RE ions are measured. As the concentration of RE ions increases, the breaking strength, modulus and toughness of organogel‐Eu fibers gradually increase, as shown in Figure 2b–d, respectively. This is because one RE ion could coordinate with three TPBI ligands and RE ions could perform as crosslinking points to increase the crosslinking density, thus enhancing the mechanical strength of the organogel matrix. The maximum breaking strength of organogel‐Eu‐0.1 could reach up to 62 MPa. However, the breaking strength, modulus, and toughness show an obvious decrease at high concentration of RE ions, for example organogel‐Eu‐0.15. In this case, excess positively charged RE ions will interact with negatively charged DNA, thus weakening the electrostatic interactions between TPBI ligands and DNA and lowering the fiber mechanical performances.

As the atomic number of RE ions increases, the breaking strength of organogel‐RE fibers also steadily increase, as shown in Figure 2e. The result is attributed to the fact that RE ions with a larger atomic number have a smaller size, thus possessing a stronger crosslinked network with strengthened coordination between RE ions and TPBI ligands. Among the organogel‐RE fibers, organogel‐Dy fibers show the highest breaking strength of ≈80 MPa, which surpasses most gel materials with a breaking strength between 0.1 and 54 MPa.^[^ ^39^, ^40^, ^41^, ^42^ ^]^ Different from the breaking strength, the modulus and toughness of organogel‐RE fibers show little dependence on the type of RE ions, as shown in Figure 2f,g, respectively.

The excellent mechanical properties of organogel‐RE fibers are attributed to the 3D crosslinked network and the alignment of DNA along the fiber direction. When organogel‐REs are stretched into organogel‐RE fibers, DNA is orientationally aligned along the stretching direction, which is confirmed by the optical images of organogel‐RE fibers under crossed polarizers, as shown in Figure 2h,i and Figures S14 and S15 in the Supporting Information. The fibers appear dark at ≈0° and become bright at ≈45°, suggesting that the fibers are birefringent and DNA strands are orientationally ordered along the fiber direction. The orientational alignment of DNA is further confirmed by 2D small angle X‐ray scattering (SAXS) patterns, which show a strong angular dependence, as demonstrated in Figure 2j and Figures S16 and S17 in the Supporting Information. The morphologies of cylindrical organogel‐RE fibers are revealed by the scanning electron microscopy (SEM) images in Figure 2k and Figures S18 and S19 in the Supporting Information, showing relatively smooth and uniform surface.

**Figure 3:**
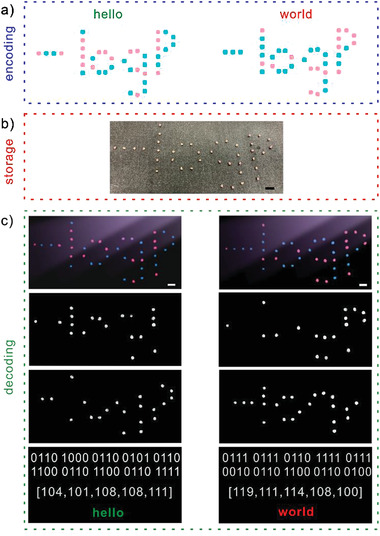
Encoding, storage, and decoding of information “hello” and “world” in self‐information patterns using luminescent organogel‐REs. a) Encoding of “hello” (left) and “world” (right) into self‐information patterns. Red and green dots in the self‐information patterns represent the binary codes of “0” and “1,” respectively. “hello” and “world” have the same apparent pattern of “−logP,” since they have the same number of characters. b) Preparation of self‐information patterns for “hello” and “world” using red and green luminescent organogel‐REs. Their patterns appear the same under regular light. c) Decoding of “hello” and “world” from the self‐information patterns under 365 nm illumination. Based on the color of each dot, binary code of “0” or “1” is extracted in series from the self‐information patterns using a customized program. The obtained binary codes are then mapped to ASCII codes to retrieve the information of “hello” and “world.” The scale bars are 1 cm.

The excellent mechanical properties and tunable luminescent colors of organogel‐REs allow the flexible design of the systems for information storage and encryption. To demonstrate this, red luminescence of organogel‐Eu and green luminescence of organogel‐Tb, which represent the binary codes of “0” and “1,” respectively, are used to encode, store and decode information “hello” and “world” in self‐information patterns of “−logP,” as shown in **Figure** **3**a. “hello” and “world” have a same number of characters and thus they could be encoded into self‐information patterns with a same apparent shape of “−logP,” as shown in Figure 3b. However, the corresponding 8‐bit ASCII codes for “hello” and “world” are encrypted in the dot colors, which could be revealed under 365 nm illumination, as shown in Figure 3c. To decipher the binary information stored in the self‐information patterns, color recognition based on the hue, saturation and value (HSV) color space and circle detection program using the Hough transform are applied to identify the dot colors and locate the dot coordinates, respectively, as shown in Figure 3c. Improved bubble sorting algorithm is then applied to sort the dot coordinates and binary codes of “0” or “1” are extracted in order based on the color of each dot. The obtained binary codes are then mapped to ASCII codes to retrieve the information of “hello” and “world.” Therefore, information “hello” and “world” could be stored and encrypted in self‐information patterns with a same apparent shape. The information could be retrieved by first imaging the patterns under 365 nm illumination and then deciphering the binary information from the color images using a customized program, greatly enhancing the flexibility and security of information storage. Therefore, security and digitalization are well addressed by our system that information could only be retrieved by decrypting the patterns and pattern fluorescent colors using the color recognition and shape detection program.

**Figure 4:**
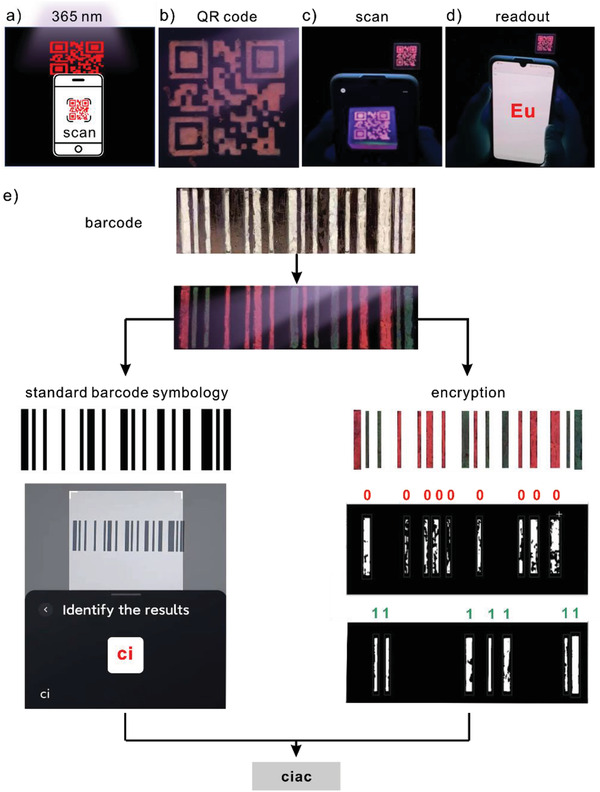
Designs of QR code and barcode using luminescent organogel‐REs for information storage and encryption. a) Schematics showing the decoding of a luminescent QR code under 365 nm illumination via a cell phone. b) The QR code prepared by organogel‐Eu appears red under 365 nm illumination. c) Scan of the QR code and d) readout of the result “Eu” via a cell phone. “Eu” is retrieved only when both the pattern and color are correct. e) Encoding and decoding of information “ciac” stored and encrypted in a barcode. Information “ci” is stored in the barcode according to Code 128B, a standard high‐density barcode symbology. Information “ac” is encrypted in the luminescent colors of the barcode stripes. Red organogel‐Eu and green organogel‐Tb stripes represent the binary codes of “0” and “1,” respectively. Complete information of “ciac” is retrieved by combining “ci” decoded from the barcode and “ac” deciphered from the colors of the barcode stripes under 365 nm illumination.

To further explore the applications of luminescent organogel‐REs as information storage media, complex information storage and encryption systems are designed and prepared. For example, the QR code for Eu is first designed according to IEC 18004 and a corresponding mold is prepared by 3D printing to mold organogel‐Eu into the predesigned pattern. By scanning the QR code under 365 nm illumination via a cell phone, information “Eu” is retrieved only when the pattern matches with the red color, as shown in **Figure** **4**a–d. In this case, information “Eu” is stored in the QR pattern while confirmation information is encrypted in the luminescent color. With dedicated design, more complex information could be encoded and encrypted in the patterns made of organogel‐REs, such as information “ciac” stored in the barcode, as shown in Figure 4e. Information “ci” is encoded in the barcode according to the standard barcode symbology, in which information is stored in the stripe width and the spacing between them. Meanwhile, information “ac” is encrypted in the stripe colors. During the decoding process, information “ci” is retrieved by scanning the barcode and mapping the results according to Code 128B. Information “ac” is deciphered from the luminescent colors of the stripes. Series of “0” and “1,” which correspond to red organogel‐Eu and green organogel‐Tb stripes, respectively, are obtained using color recognition based on the HSV color space and stripe detection program based on the image contour coding. The binary codes are then mapped to ASCII codes to retrieve information “ac.” Complete information “ciac” is retrieved by combining “ci” obtained from the standard barcode symbology and “ac” encrypted in the color of the barcode stripes.

**Figure 5:**
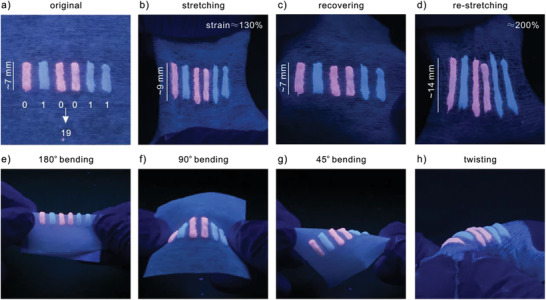
Information storage on a flexible substrate using luminescent organogel‐REs. a) Encoding of number “19” in a binary pattern on a flexible substrate, in which red organogel‐Eu stripe and green organogel‐Tb stripe represent the binary codes of “0” and “1,” respectively. b) Stretching, c) recovering, d) restretching, e) 180° bending, f) 90° bending, g) 45° bending, and h) twisting of the binary pattern, showing excellent stretchability, bendability, and stability. Organogel‐REs are solvated with DMSO.

Organogel‐REs are good candidates of information storage media and they possess advantages of tunable fluorescent color, good processability, high stretchability, and excellent stability. Combined with the customized color recognition and shape detection program, various self‐information patterns could be designed for information storage and encryption and could be used in different scenarios, such as on flexible substrates. Owing to the plasticity of the organogel‐REs, the pattern will deform as the substrate deforms and recover as the substrate recovers. Therefore, the information pattern will not be damaged after deformations. For example, encoding of number “19” in a binary pattern on a flexible substrate, in which red organogel‐Eu stripe and green organogel‐Tb stripe represent the binary codes of “0” and “1,” respectively, could maintain its integrity under repeating actions of high‐magnitude stretching, bending, and twisting, as shown in **Figure** **5**a–h. After the rude operations, the information could correctly be retrieved from the luminescent colors of the patterns, which displayed great potentials of luminescent organogel‐REs for information storage and encryption on flexible devices.

Our work is the first example to demonstrate the excellent mechanical properties of fluorescent materials and the flexible design of self‐information patterns facilitated by a customized color recognition and shape detection program. Organogel‐REs possess advantages of tunable fluorescent color, good processability, high stretchability, and excellent stability, which allow the encryption of information in various self‐information patterns and maintain their integrity under repeated deformations of various scenarios. Pattern colors are revealed under UV light. Information could only be retrieved by decrypting the patterns and pattern colors together using the color recognition and shape detection program.

## 3 Conclusion

A series of new lanthanide‐based organogels showing different luminescent colors are developed for information storage and encryption. The organogels are prepared by electrostatic and coordinative interactions between natural DNA, synthetic ligands and rare earth ions. Due to the noncovalent interactions in the gel matrix, the organogel‐REs could be stretched by 180 times and possess excellent mechanical performances with a breaking strength up to 80 MPa, which are superior over most reported gel materials. By utilizing the luminescent colors of organogel‐REs as the binary codes of “0” and “1” for data encoding and decoding, a series of applications are realized, including self‐information pattern, QR code and barcode, which implement both information storage and encryption. Decoding of information stored in the apparent patterns and deciphering of information encrypted in the luminescent colors are greatly facilitated by a customized color recognition and shape detection program. The unique properties of luminescent organogel‐REs, such as easy for processing, simple for information encoding, stable for information storage, and safe for information decoding, make the systems promising for information storage and encryption in multi scenarios.

## Conflict of Interest

The authors declare no conflict of interest.

## Supporting information

Supporting Information

## Data Availability

The data that support the findings of this study are available from the corresponding author upon reasonable request.
