# Supplementary material for: Highly Plasticized Lanthanide Luminescence for Information Storage and Encryption Applications
Source: Adv Sci (Weinh). 2022 Jan 12;9(7):2105108. doi: 10.1002/advs.202105108 (PMC8895122; doi:10.1002/advs.202105108)
Supplement: Supplementary file 1 — Supporting Information [file ADVS-9-2105108-s001.pdf]

WILEY-VCH

ADVANCED SCIENCE
Open Access

Supporting Information

for *Adv. Sci.*, DOI: 10.1002/advs.202105108

Highly Plasticized Lanthanide Luminescence for Information Storage and Encryption Applications

Yawei Liu, Kelu Zhao, Yubin Ren, Sikang Wan, Chenjing Yang, Jingjing Li, Fan Wang, Chunying Chen, Juanjuan Su, Dong Chen*, Yuliang Zhao, Kai Liu*, and Hongjie Zhang*

Supporting Information

Highly Plasticized Lanthanide Luminescence for Information Storage and Encryption Applications

Yawei Liu#, Kelu Zhao#, Yubin Ren, Sikang Wan, Chenjing Yang, Jingjing Li, Fan Wang, Chunying Chen, Juanjuan Su, Dong Chen*,Yuliang Zhao, Kai Liu* and Hongjie Zhang*

Table of Contents

1. Experimental Materials and Methods. 3
2. Figures 6
3. Codes 15
4. References 26

1. Experimental Materials and Methods.

Materials. 3,5-dimethoxybenzaldehyde (99.60%), 1-(pyridin-2-yl)ethanone (98%) and 1,6-dibromohexane (97%) were purchased from Shanghai Bide Pharmaceutical Technology Co., Ltd. Lanthanum(III) chloride hexahydrate ($LaCl_3·6H_2O$, 99.99%), Cerium (III) chloride hexahydrate ($CeCl_3·6H_2O$, 99.99%), Samarium (III) chloride hexahydrate ($SmCl_3·6H_2O$, 99.99%), Europium chloride hexahydrate ($EuCl_3·6H_2O$, 99.99%), Terbium (III) chloride hexahydrate ($TbCl_3·6H_2O$, 99.99%), and Dysprosium (III) chloride hexahydrate ($DyCl_3·6H_2O$, 99.99%) were purchased from Saen Chemical Technology Co., Ltd. Anhydrous ethanol, ammonia solution (25%), 1-methylimidazole (99%), dimethyl sulfoxide (DMSO, AR) and bromic acid (40%) was purchased from Shanghai Macklin Biochemical Co., Ltd. N-hexane (97.0%), ethyl acetate (EtOAc, 99.5%), acetonitrile (CH_3CN, 99.0%), sodium bicarbonate ($NaHCO_3$, 99.5%), potassium carbonate (K_2CO_3, 99.5%) and potassium hydroxide (KOH, 90%) were purchased from Shanghai TITAN Technology Co., Ltd. All deuterated solvents ($CDCl_3$ and DMSO-d6) were purchased from Cambridge Isotope Laboratories, Inc. The salmon sperm DNA with a molecular weight of ~1300 KDa and around 2000 base-pairs was purchased from Dingguo Changsheng Biotechnology Co., Ltd.

Synthesis of 4'-(3,5-dimethoxyphenyl)-2,2':6',2''-terpyridine (denoted as compound 1). The compound 1 was synthesized according to reported procedures.[1] Briefly, 3,5-dimethoxybenzaldehyde (3.32 g, 20 mmol) and 1-(pyridin-2-yl)ethanone (4.84 g, 40 mmol) were added into a 250 mL flask with 100 mL anhydrous ethanol, followed by an addition of KOH (3.08 g, 35 mmol). The mixture was stirred under N_2 atmosphere for 12 h at room temperature. After adding 60 mL ammonia solution (25%) into the above-mentioned mixture dropwise, the whole mixture was refluxed for 24 h. Then the mixture was filtered and the residue was washed with 300 mL anhydrous ethanol. The dry crude product was further purified by flash column chromatography on silica with the elution of EtOAc and n-hexane (4:6, v:v). Finally, white powdery compound 1 (5.17 g, 70%) was obtained.
^1H NMR (400 MHz, $CDCl_3$, 25 °C) δ 8.73 (d, 2H), 8.70 (s, 2H), 8.67 (d, 2H), 7.88 (t, 2H), 7.36 (t, 2H), 7.01 (s, 2H), 6.56 (s, 1H), 3.90 (s, 6H).
^{13}C NMR (100 MHz, $CDCl_3$, 25 °C) δ 161.24, 156.10, 150.52, 149.15, 140.87, 136.88, 123.84, 121.41, 119.14, 105.56, 101.14, 55.64.

Synthesis of 5-([2,2':6',2''-terpyridin]-4'-yl)benzene-1,3-diol (denoted as compound 2). The mixture of compound 1 (3.69 g, 10 mmol) and 80 mL bromic acid (40%) was refluxed at 130 ℃ overnight. After removing excess bromic acid, yellow solid was obtained. Then the yellow solid was washed with saturated $NaHCO_3$ solution until the yellow color faded. Afterwards, white compound 2 (2.73 g, 80%) was obtained after filtration and dehydration process.
^1H NMR (400 MHz, DMSO-d6, 25 °C) δ 8.67 (m, 6H), 8.04 (s, 2H), 7.53 (s, 2H), 6.78 (s, 2H), 6.36 (s, 2H).
^{13}C NMR (100 MHz, DMSO-d6, 25 °C) δ 160.22, 156.01, 155.43, 150.16, 149.84, 139.42, 137.94, 124.98, 121.32, 118.06, 105.00, 104.35.

Synthesis of 4'-(3,5-bis((6-bromohexyl)oxy)phenyl)-2,2':6',2''-terpyridine (denoted as compound 3). A mixture of 1,6-dibromohexane (12.1g, 50 mmol), K_2CO_3 (1.38 g, 10 mmol) and 100 mL anhydrous CH_3CN was stirred and heated to 70 ℃. Then, 100 mL CH_3CN suspension with compound 2 (1.7g, 5 mmol) was added dropwise into above solution.

The whole mixture was refluxed for 24 h. Compound 3 was attained after filtration, concentration and purification by flash column chromatography on silica with the elution of EtOAc and n-hexane (3:7, v:v) (1.8 g, 54%).
^{1}H NMR (400 MHz, $CDCl_3$, 25 °C) δ 8.73 (d, 2H), 8.69 (s, 2H), 8.67 (d, 2H), 7.88 (t, 2H), 7.36 (t, 2H), 7.00 (s, 2H), 4.05 (t, 4H),1.92 (m, 4H), 1.84 (m, 4H), 1.54 (m, 8H).
^{13}C NMR (100 MHz, $CDCl_3$, 25 °C) δ 160.68, 156.31, 155.91, 150.53, 149.14, 140.72, 136.88, 123.83, 121.43, 119.09, 106.16, 101.85, 68.02, 33.83, 32.73,29.17, 27.98, 25.36.

Synthesis of 1,1'-(((5-([2,2':6',2''-terpyridin]-4'-yl)-1,3-henylene)bis(oxy))bis (hexane-6,1-diyl))bis(3-methyl-1H-imidazol-3-ium) (denoted as TPBI). A mixture of 1-methylimidazole (0.82 g, 10 mmol), compound 3 (1 g, 1.5mmol) and 100 mL CH_3CN was refluxed for 24 h. After removing CH_3CN, recrystallization was implemented by using the mixture of extensive ethyl acetate and 3 mL deionized water repeatedly to attain TPBI powders (0.643 g, 64%).
^{1}H NMR (400 MHz, DMSO-d6, 25 °C) δ 9.23 (s, 2H), 8.77 (d, 2H), 8.68 (d, 2H), 8.64 (s, 2H), 8.06 (t, 2H), 7.83 (s, 2H), 7.74 (s, 2H),7.56 (t, 2H), 6.96 (s, 2H), 6.64 (s, 1H), 4.21 (t, 4H), 4.09 (s, 4H), 3.87 (s, 6H), 1.85 (m, 4H), 1.76 (m, 4H), 1.50 (m, 4H), 1.35 (m, 4H).
^{13}C NMR (100 MHz, DMSO-d6, 25 °C) δ 161.04, 156.12, 155.40, 150.18, 149.83, 140.44, 138.05, 136.99, 125.10, 124.09, 122.76, 121,52, 118.74, 105.83, 102.55, 68.13, 49.21, 36.23, 29.82, 28.98, 25.76, 25.44.

Fabrication of organgel-REs. DNA, TPBI and lanthanide ions were mixed together in aqueous solution firstly. The adding amount of DNA and TPBI were calculated according their electric charges. Here, 1 mL DNA solution (10 $mg \cdot mL^{-1}$) and 1 mL TPBI solution (10 $mg \cdot mL^{-1}$) was mixed together to guarantee the nearly equal charge of DNA and TPBI. The adding amount of lanthanide ions was calculated according the coordination between TPBI and lanthanide.[2] Three TPBI ligands could coordinate with one RE ion. Thus, 0, 4.8, 24, 48 and 72 μL $EuCl_3$ solution was added in the mixture with a mole ratio of Eu to TPBI = 0:3, 0.01:3, 0.05:3, 0.1:3 and 0.15:3, which denotes as organogel-Eu-0, organogel-Eu-0.01, organogel-Eu-0.05, organogel-Eu-0.10 and organogel-Eu-0.15, respectively. Besides, other lanthanide chloride solution including $LaCl_3$, $CeCl_3$, $SmCl_3$, $TbCl_3$ and $DyCl_3$ was also mixed with a mole of REs to TPBI = 0.1:3 to generate organogel-REs, which denoted as organogel-La, organogel-Ce, organogel-Sm, organogel-Tb and organogel-Dy, respectively. After the mixing process, white deposit occurred immediately. The deposition was centrifugated at a rate of 12000 r/min for 2 min. Then the precipitate was washed with deionized water. The above circle was repeated 2 times. Finally, the collected sediment was lyophilized and then swelled with three droplets of DMSO overnight to form organgel-REs.

Fabrication of organgel-RE fibers. The organogel-RE was kneaded and stretched manually before use. To generate organogel-RE fibers, a thin stainless steel rod was vertically dipped into the organogel-REs and drawn out at a speed of 2 cm/s. Organogel-REs was attached on the rod and stretched to organogel-RE fibers during this process. Organogel-RE fibers were collected continuously by twining on a nylon framework. The organogel-RE fibers were stored in room temperature for 1 hour for further test. The organogel-RE fibers made of organogel-Eu-0, organogel-Eu-0.01, organogel-Eu-0.05 organogel-Eu-0.1, organogel-Eu-0.15, organogel-La, organogel-Ce, organogel-Sm, organogel-Tb and organogel-Dy were denoted as organogel-Eu-0 fiber, organogel-Eu-0.01 fiber, organogel-Eu-0.05 fiber, organogel-Eu-0.1 fiber, organogel-Eu-0.15 fiber, organogel-La fiber, organogel-Ce fiber, organogel-Sm fiber, organogel-Tb fiber and organogel-Dy fiber, respectively.

Measurements. The tensile test of various organogel-RE fibers was implemented on the FAVIMAT+ instrument (Textechno, German) with a 2 N load cell at a speed of 5 mm·min^{-1} at room temperature and a gauge length of 2 mm was maintained between the clamps. Breaking strength was calculated according to the following formula of breaking strength=$F/\pi r^2$. While, F was axial breaking force that number could be obtained through test instrument. And r was the radius of the organogel-RE fibers, which obtained by measuring organogel-RE fibers under microscopy. The internal molecular orientation of organogel-RE fibers were assessed by a polarizing microscope (Nikon, ECLIPSE LV100N POL, 100-240 V, 1.2 A, 50/60 Hz). Morphology observation of the surface of the organogel-RE fibers was performed by ZEISS scanning electron microscopy at 1 kV. Synchrotron Radiation Small Angle X-ray Scattering (SAXS) analysis of the organgel-RE fiber was carried out in BL19U2 station of Shanghai Synchrotron Radiation Facility (SSRF). The chosen X-ray wavelength (λ) was 0.923 nm (energy was 13.43 keV). The sample-to-detector distance was 2336 mm. The exposure time for each organogel-RE fiber measurement was ranged from 5-10 s. The single organogel-RE fiber was strengthened and mounted onto a hollow specimen holder by double-sided adhesive tape, and then put on the test holder with the fiber vertical. ^{1}H and ^{13}C NMR were recorded on a Bruker AVANCE III 400 NMR spectrometer. Mass spectrometry was collected on Quattro Premier XE with ESI source. Thermogravimetric (TG) test was implemented using a netzsch STA449F3 with an automated vertical overhead thermobalance. Samples were heated from room temperature to 800 °C at a rate of 10 °C/min under N_2 atmosphere.

2. Figures

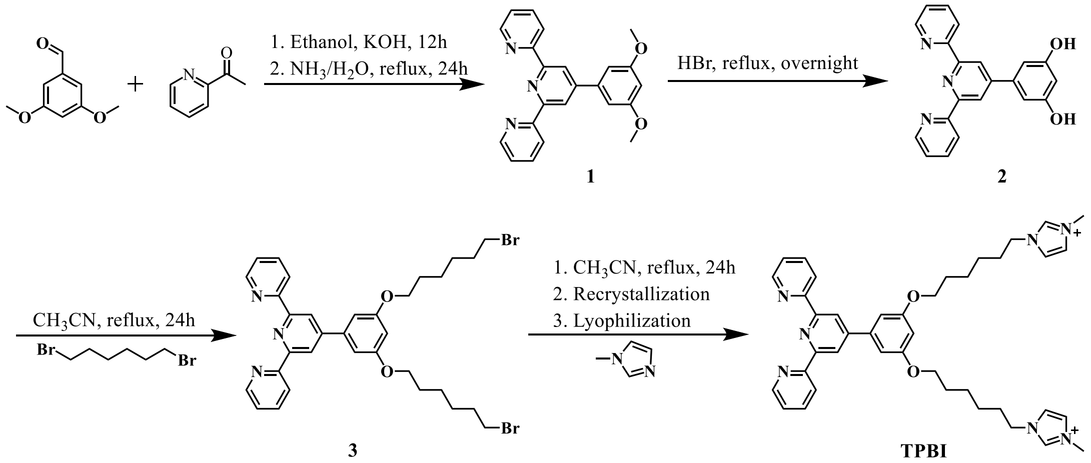

Figure S1: Synthetic route of TPBI.

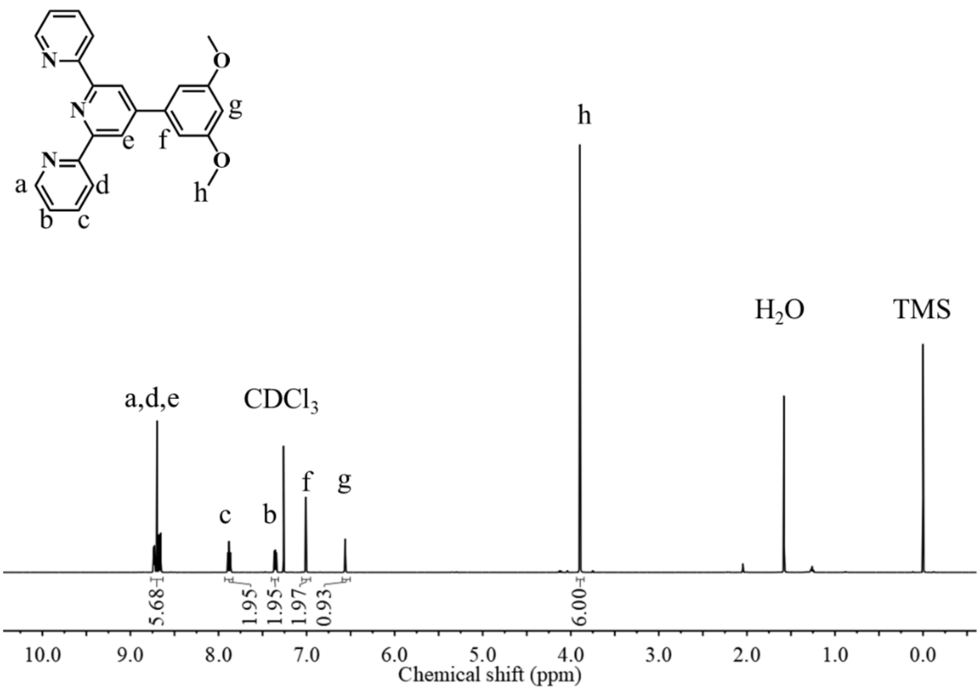

Figure S2: ^{1}H NMR spectrum of **compound 1**. Frequency=400 MHz; Solvent=$CDCl_3$; Temperaure=25 °C.

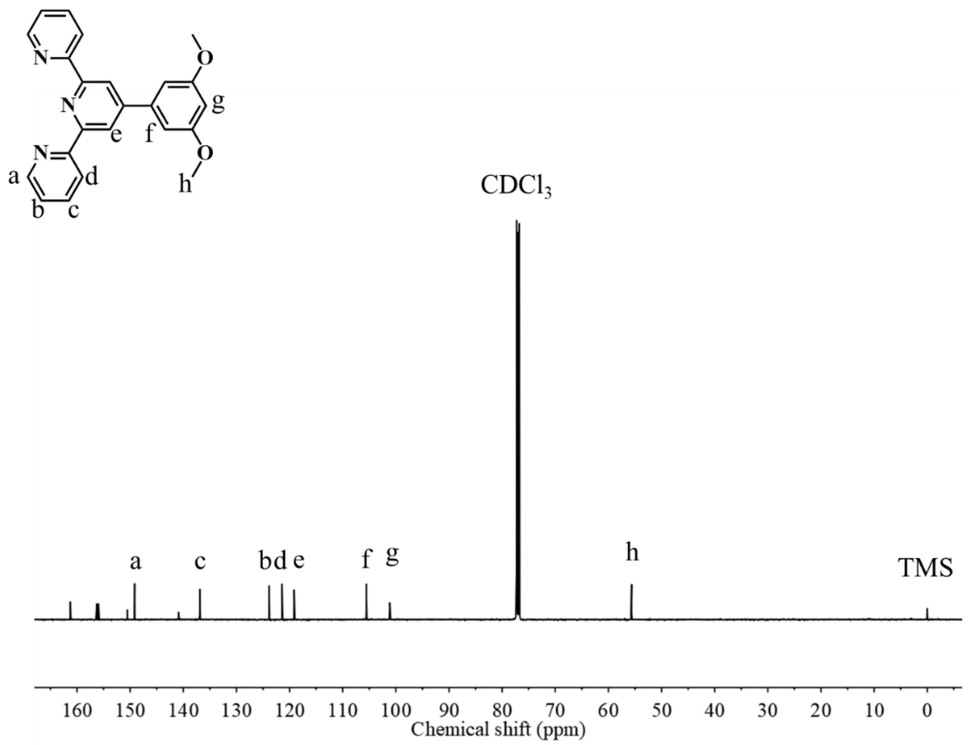

Figure S3: ^{13}C NMR spectrum of **compound 1**. Frequency=400 MHz; Solvent=$CDCl_3$; Temperaure=25 °C.

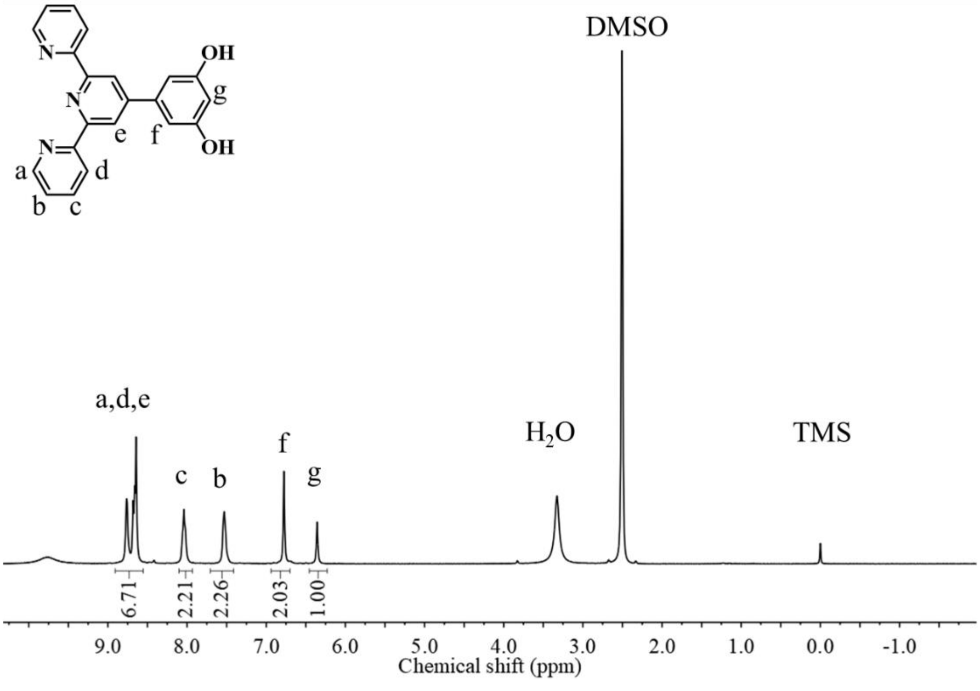

Figure S4: ^{1}H NMR spectrum of **compound 2**. Frequency=400 MHz; Solvent=DMSO-d6; Temperaure=25 °C.

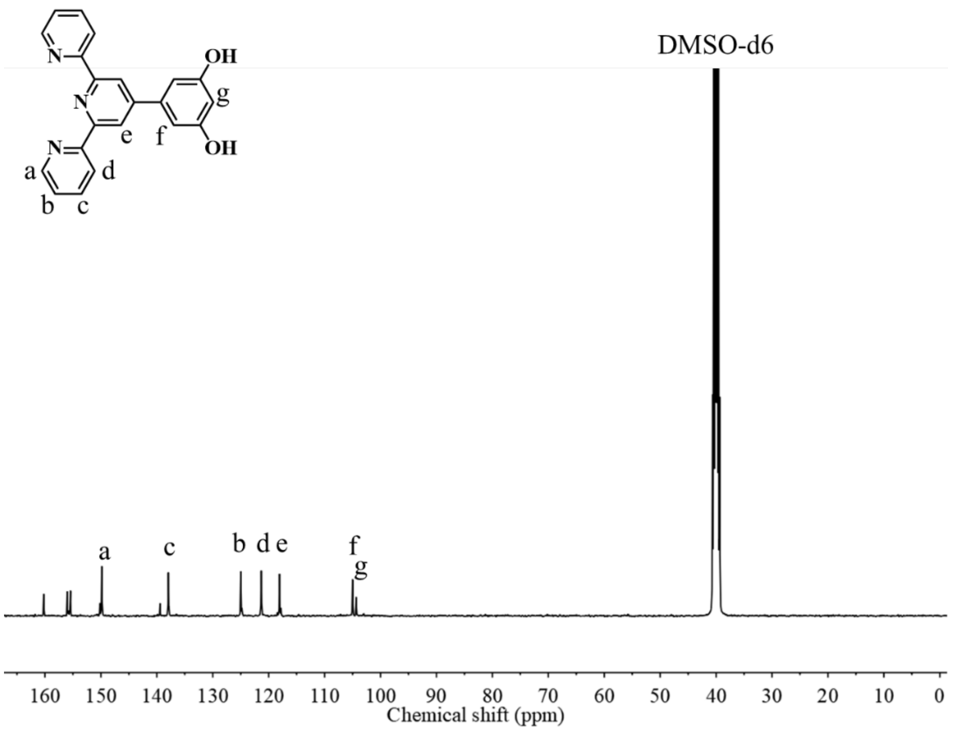

Figure S5: ^{13}C NMR spectrum of **compound 2**. Frequency=400 MHz; Solvent=DMSO-d6; Temperaure=25 °C.

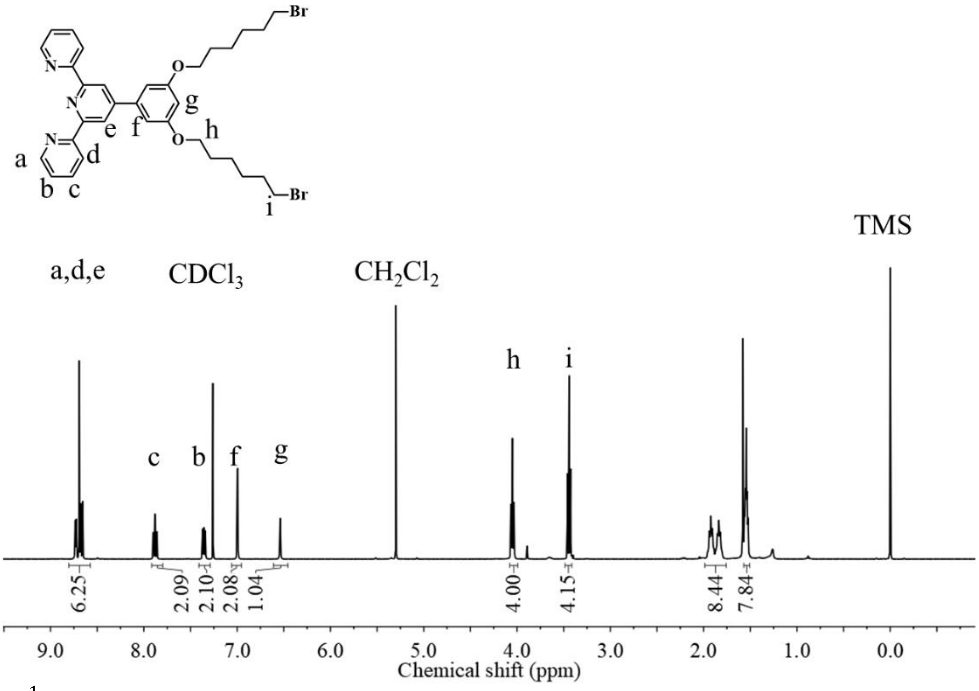

Figure S6: ^{1}H NMR spectrum of **compound 3**. Frequency=400 MHz; Solvent=$CDCl_3$; Temperaure=25 °C.

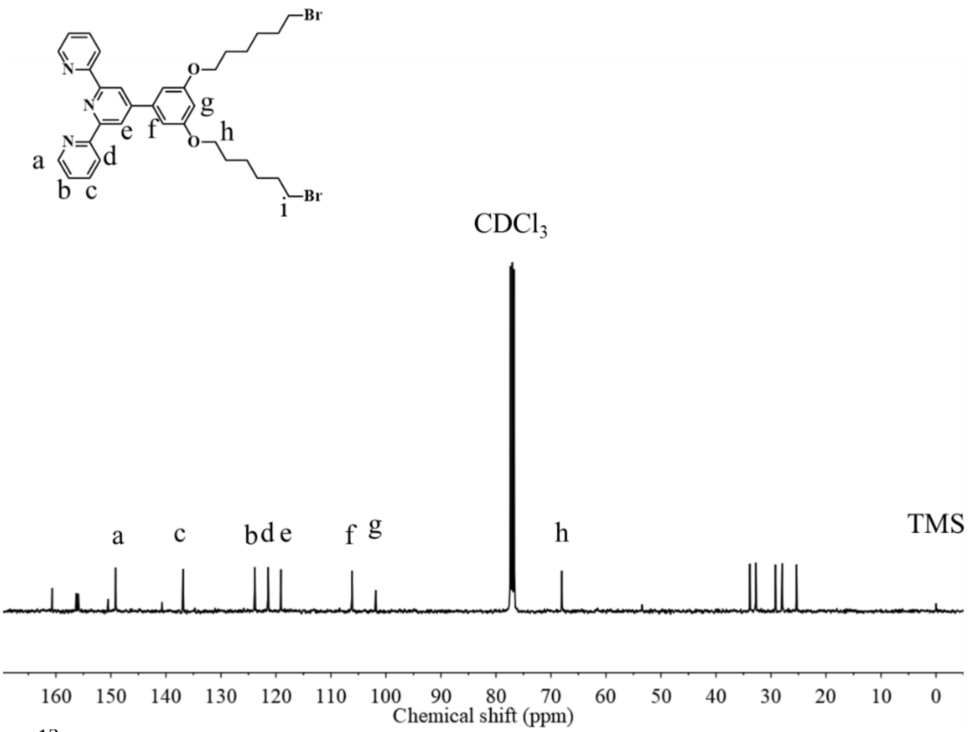

Figure S7: ^{13}C NMR spectrum of **compound 3**. Frequency=400 MHz; Solvent=$CDCl_3$; Temperaure=25 °C.

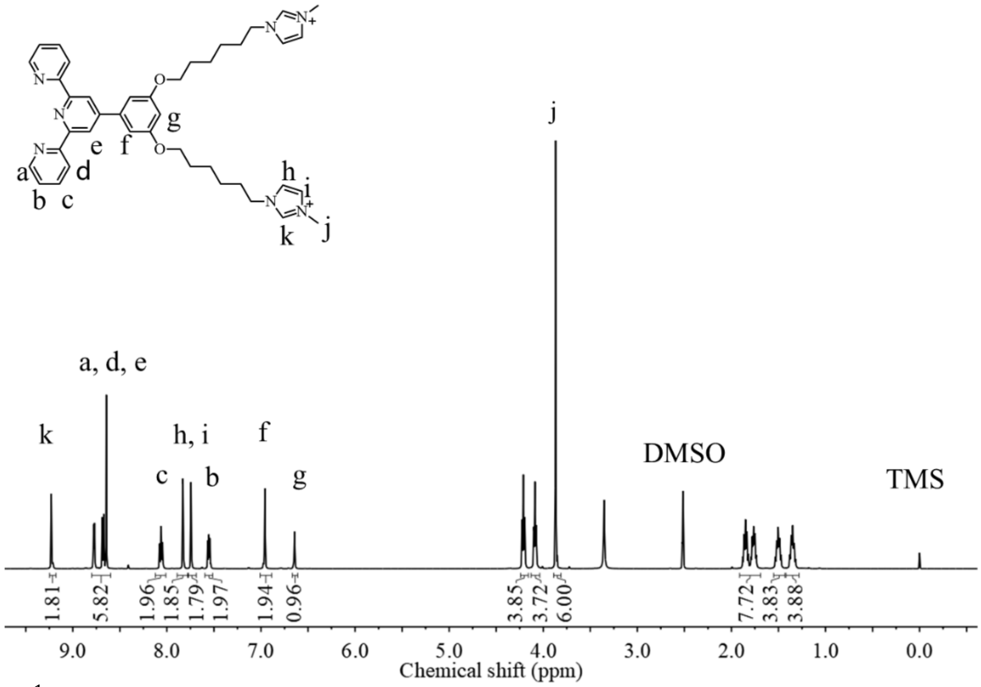

Figure S8: ^{1}H NMR spectrum of TPBI. Frequency=400 MHz; Solvent=DMSO-d6; Temperaure=25 °C.

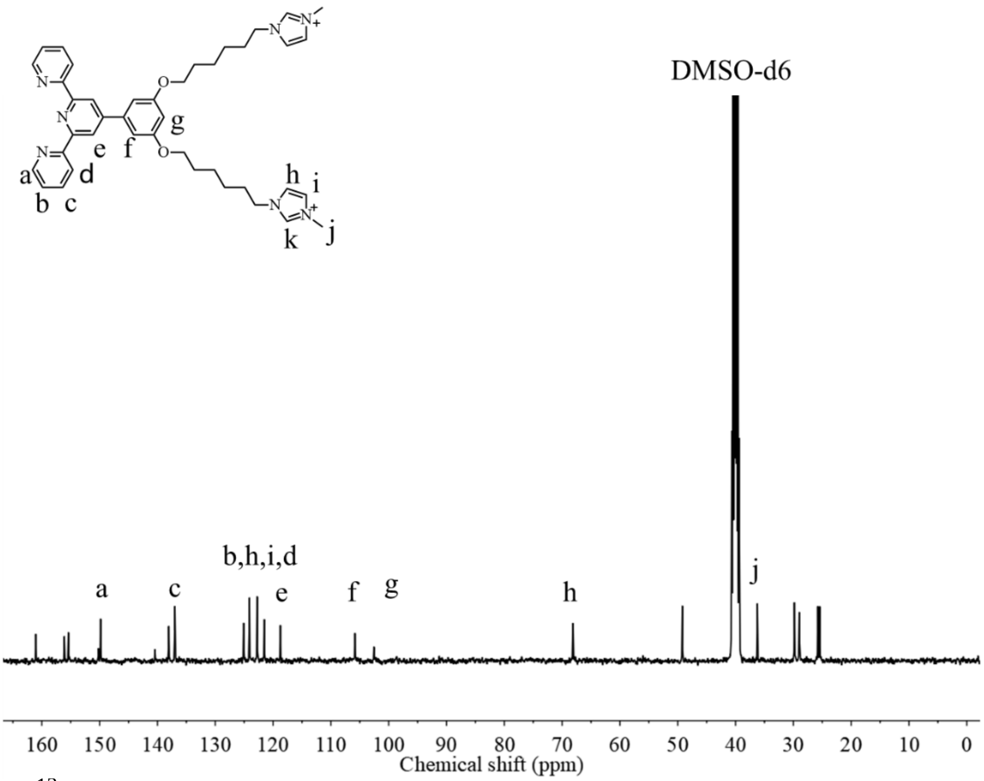

Figure S9: ^{13}C NMR spectrum of TPBI. Frequency=400 MHz; Solvent=DMSO-d6; Temperaure=25 °C.

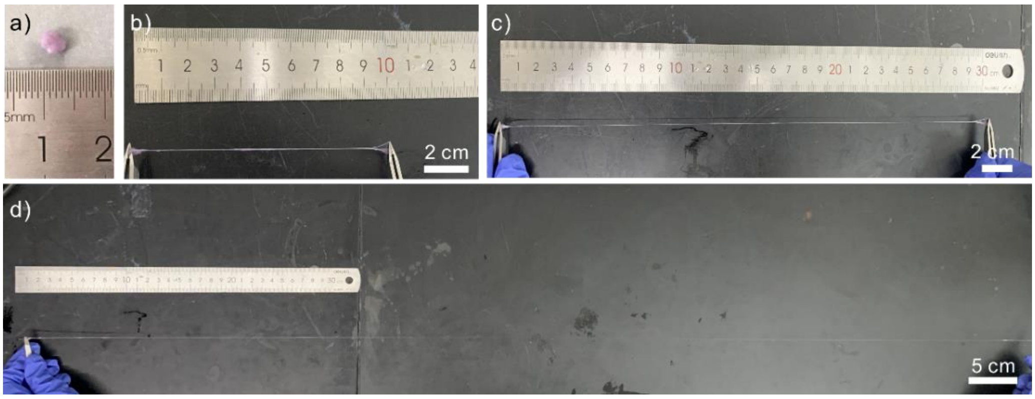

Figure S10: a) Unstretched organogel without lanthanide. b), c) and d) Organogel fiber stretched by 20, 60 and 180 times, respectively. The formation of DNA organogel in the absence of RE ions suggests the successful electrostatic interaction between DNA and TPBI ligands.

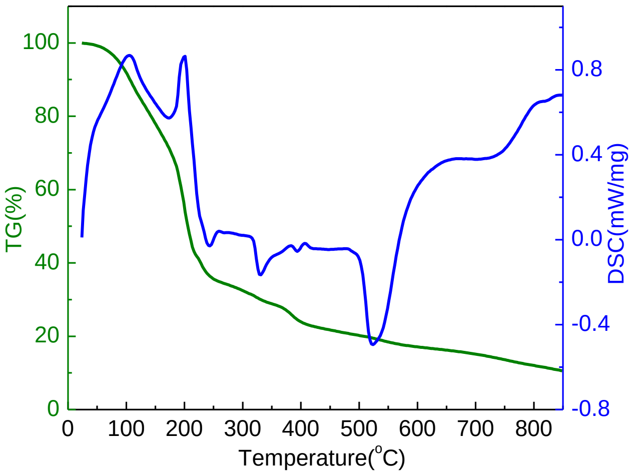

;

Figure S11: TG and DSC results of the organogel without lanthanide.

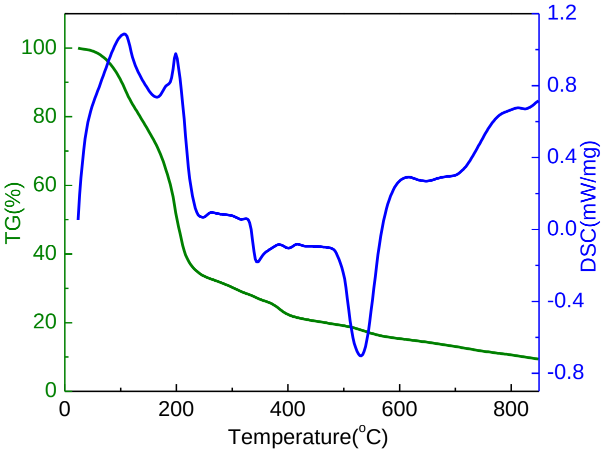

Figure S12: TG and DSC results of organogel-Eu-0.1. The mole ratio of Eu to TPBI = 0.1:3.

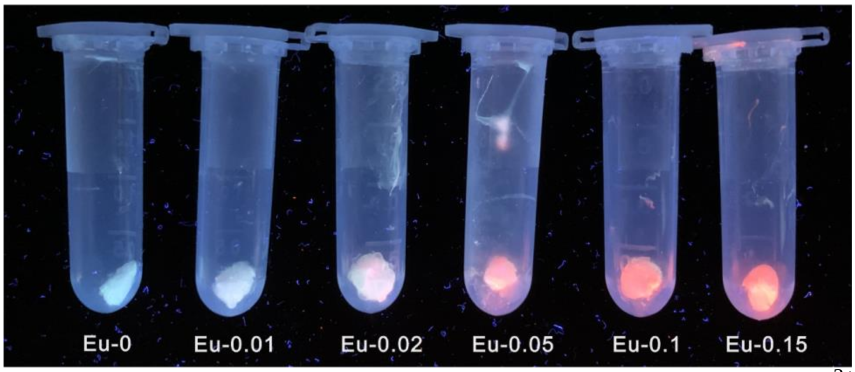

Figure S13: Luminescence of organogel-Eu with different concentration of Eu^{3+} ions. The color of organogel-Eu becomes redder as the Eu concentration increases.

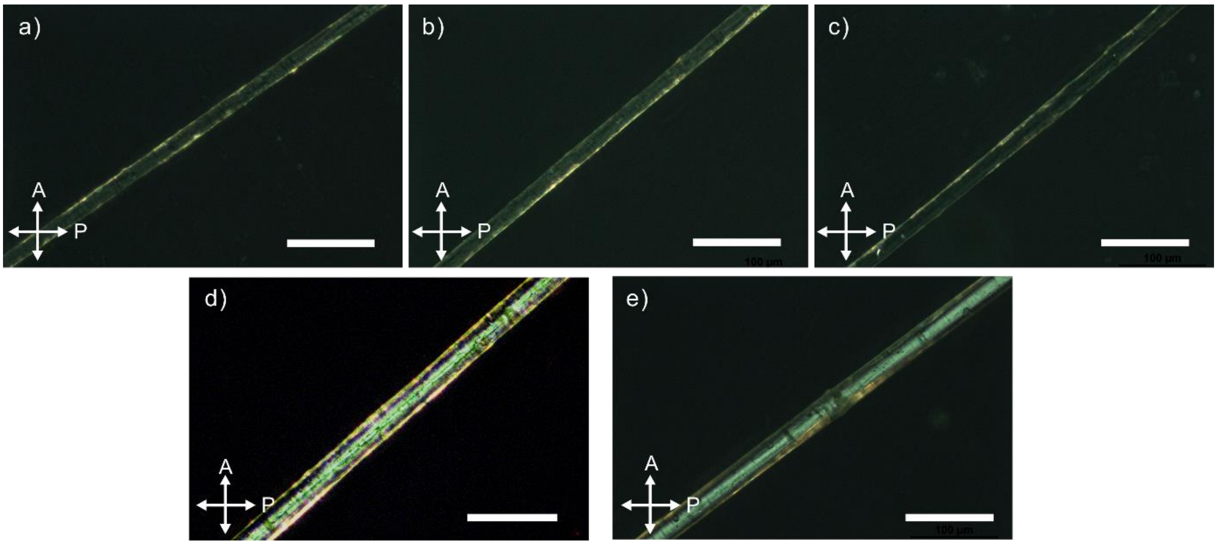

Figure S14: Optical images of organogel-Eu fibers with different concentrations of Eu under crossed polarizer and analyzer. a) Eu-0, b) Eu-0.01, c) Eu-0.05, d) Eu-0.1 and e) Eu-0.15 fibers with the mole ratio of Eu to TPBI ligand = 0, 0.01:3, 0.05:3, 0.1:3 and 0.15:3, respectively. The concentration of TPBI ligands is fixed at 5 mg·mL^{-1}. The scale bars are 100 µm.

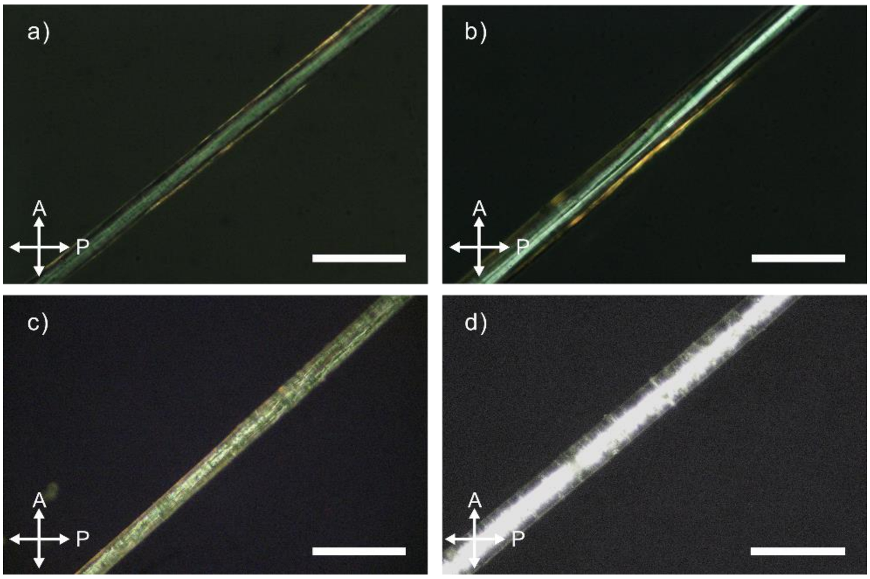

Figure S15: Optical images of organogel-RE fibers with different lanthanides under crossed polarizer and analyzer: a) organogel-La fiber, b) organogel-Ce fiber, c) organogel-Sm fiber and d) organogel-Tb fiber. The scale bars are 100 µm.

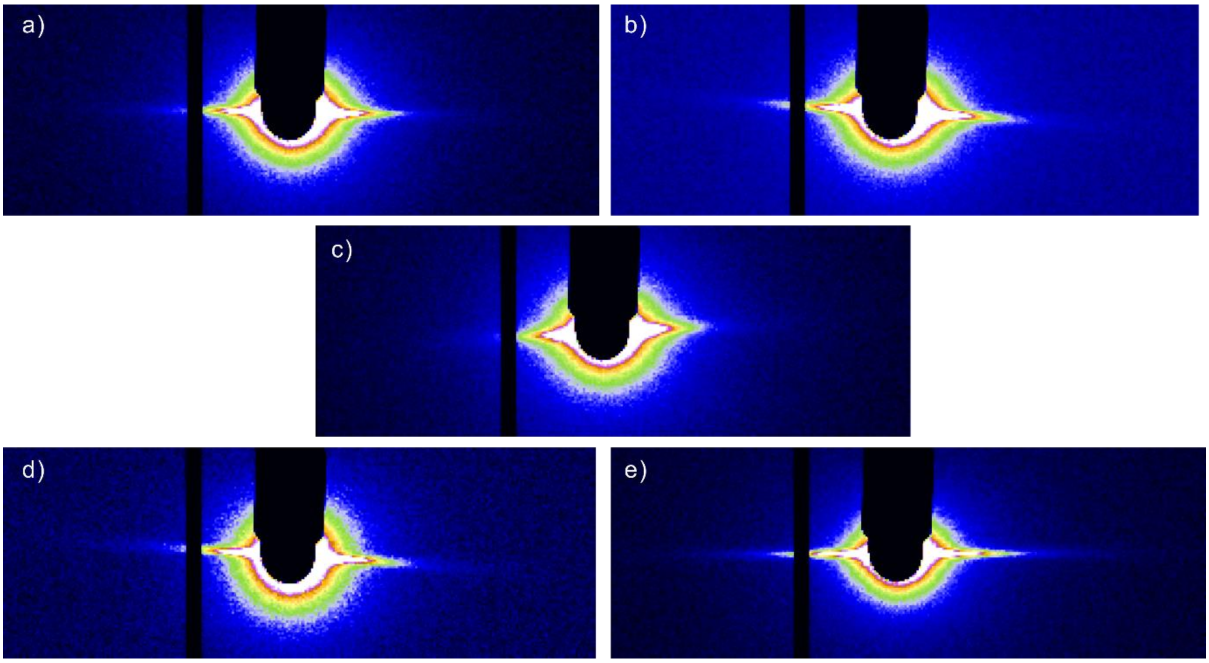

Figure S16: 2D SAXs of organogel-Eu fibers with different concentrations. a) Eu-0, b) Eu-0.01, c) Eu-0.05, d) Eu-0.1 and e) Eu-0.15 fibers with the mole ratio of Eu to TPBI ligand = 0, 0.01:3, 0.05:3, 0.1:3 and 0.15:3, respectively. The concentration of TPBI ligand is fixed at 5 $mg{\cdot}mL^{-1}$.

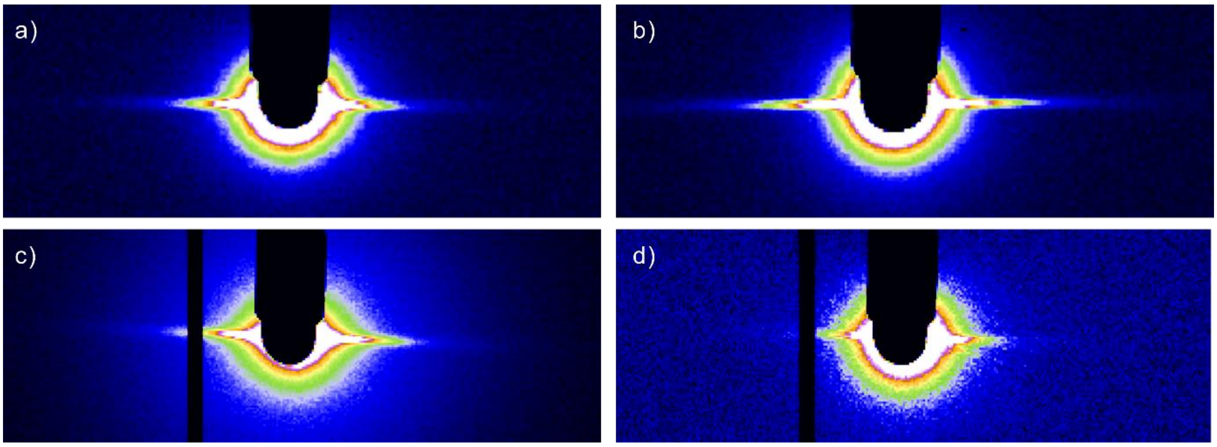

Figure 17: 2D SAXs of of organogel-RE fibers with different lanthanides: a) organogel-La fiber, b) organogel-Ce fiber, c) organogel-Sm fiber and d) organogel-Tb fiber.

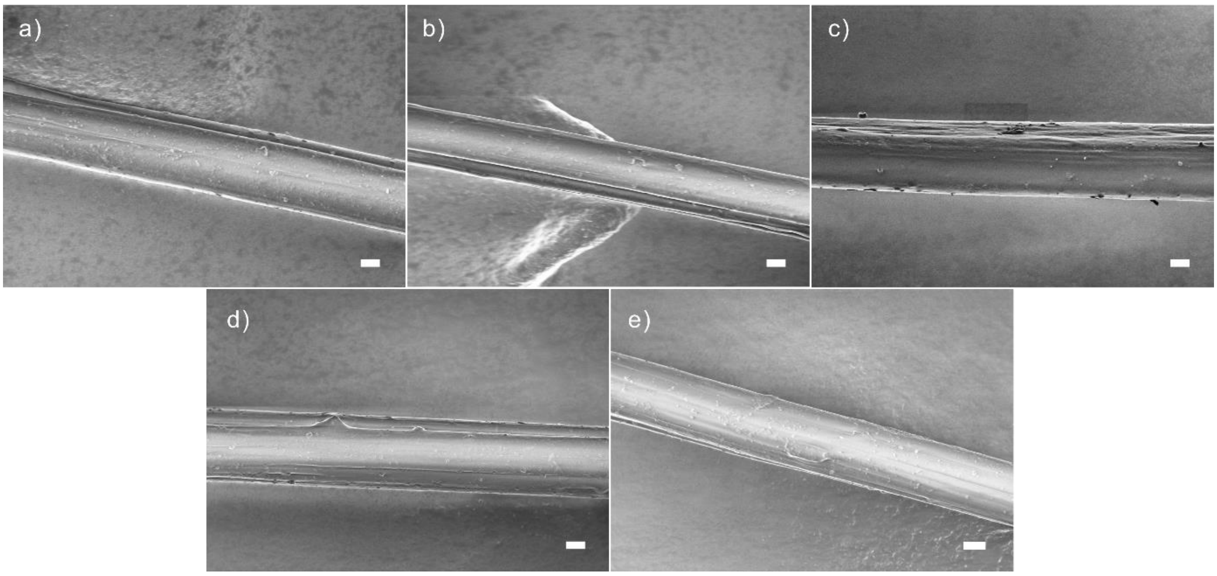

Figure S18: SEM images of organogel-Eu fibers with different concentrations. a) Eu-0, b) Eu-0.01, c) Eu-0.05, d) Eu-0.1 and e) Eu-0.15 fibers with the mole ratio of Eu to TPBI ligand = 0, 0.01:3, 0.05:3, 0.1:3 and 0.15:3, respectively. The concentration of TPBI ligands is fixed at 5 mg·mL^{-1}. The scale bars are 10 µm.

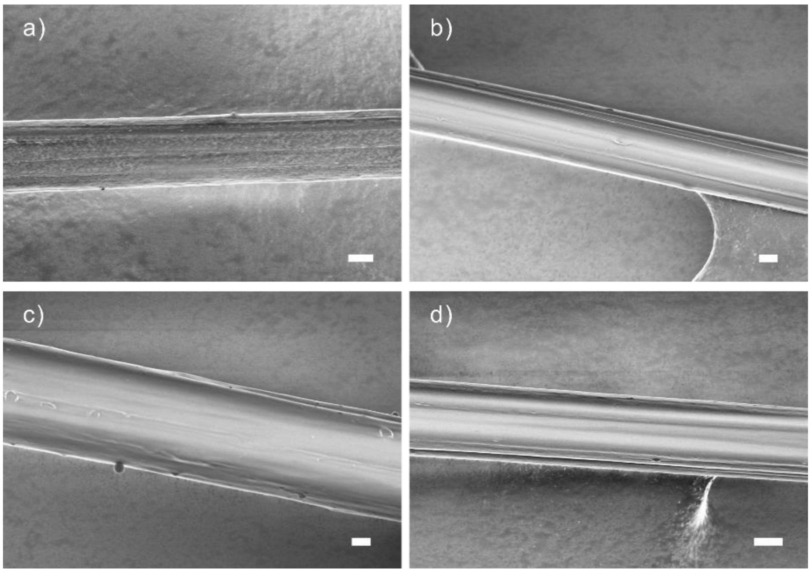

Figure S19: SEM images of organogel-RE fibers with different lanthanides: a) organogel-La fiber, b) organogel-Ce fiber, c) organogel-Sm fiber and d) organogel-Tb fiber. The scale bars are 10 µm.

3. Codes

The code for encoding “hello” and “world”.

```
from turtle import *
#minus sign
def draw_minus_sign(x,y,data,line_len=4):
    penup()
    goto(x,y)
    setheading(0)
    for i in range(line_len):
        if data[i]==0:
            dot(8,'pink')
            penup()
            forward(12)
        elif data[i]==1:
            dot(8,'cyan')
            penup()
            forward(12)
    return data[line_len:]

# l
def draw_l(x,y,data,line_len=6):
    penup()
    goto(x,y)
    setheading(-90)
    for i in range(line_len):
        if data[i] == 0:
            dot(8, 'pink')
            penup()
            forward(13)
        elif data[i] == 1:
            dot(8, 'cyan')
            penup()
            forward(13)
    return data[line_len:]

#o
def draw_o(x,y,data,line_len=8):
    penup()
    goto(x,y)
    setheading(-90)
    # the first column
    for i in range(2):
        if data[i] == 0:
            dot(8, 'pink')
            penup()
            forward(14)
        elif data[i] == 1:
            dot(8, 'cyan')
            penup()
```

```
            forward(14)
    #the second column
    goto(x+10,y+10)
    pendown()
    for i in range(2,4):
        if data[i] == 0:
            dot(8, 'pink')
            penup()
            forward(32)
        elif data[i] == 1:
            dot(8, 'cyan')
            penup()
            forward(32)
    #the third column
    goto(x + 20, y + 10)
    pendown()
    for i in range(4, 6):
        if data[i] == 0:
            dot(8, 'pink')
            penup()
            forward(32)
        elif data[i] == 1:
            dot(8, 'cyan')
            penup()
            forward(32)
    # the fourth column
    goto(x+30, y)
    pendown()
    for i in range(6, 8):
        if data[i] == 0:
            dot(8, 'pink')
            penup()
            forward(14)
        elif data[i] == 1:
            dot(8, 'cyan')
            penup()
            forward(14)
    return data[line_len:]

#g
def draw_g(x,y,data,line_len):
    penup()
    goto(x,y)
    setheading(-90)
    #the first column
    goto(x,y-8)
    pendown()
    for i in range(1):
        if data[i] == 0:
            dot(8, 'pink')
            penup()
```

```
    elif data[i] == 1:
        dot(8, 'cyan')
        penup()

#the second column
goto(x + 10, y + 7)
pendown()
for i in range(1,3):
    if data[i]==0:
        dot(8,'pink')
        penup()
        forward(29)
    elif data[i]==1:
        dot(8,'cyan')
        penup()
        forward(29)
forward(8)
pendown()
for i in range(3,4):
    if data[i]==0:
        dot(8,'pink')
        penup()
    elif data[i]==1:
        dot(8,'cyan')
        penup()

#the third column
goto(x+21,y+8)
pendown()
for i in range(4,6):
    if data[i]==0:
        dot(8,'pink')
        penup()
        forward(30)
    elif data[i]==1:
        dot(8,'cyan')
        penup()
        forward(30)
forward(10)
pendown()
for i in range(6, 7):
    if data[i] == 0:
        dot(8, 'pink')
        penup()
    elif data[i] == 1:
        dot(8, 'cyan')
        penup()
#the fourth column
goto(x+33,y)
pendown()
for i in range(7,line_len):
```

```
        if data[i] == 0:
            dot(8, 'pink')
            penup()
            forward(14)
        elif data[i] == 1:
            dot(8, 'cyan')
            penup()
            forward(14)
    return data[line_len:]

#P
def draw_p(x,y,data,line_len=10):
    avg=line_len//5
    penup()
    goto(x,y)
    setheading(-90)
    #the first column
    for i in range(avg*3):
        if data[i]==0:
            dot(8,'pink')
            penup()
            forward(13)
        elif data[i]==1:
            dot(8,'cyan')
            penup()
            forward(13)
    #the second column
    goto(x+12,y)
    pendown()
    for i in range(avg*3,avg*4):
        if data[i]==0:
            dot(8,'pink')
            penup()
            forward(28)
        elif data[i]==1:
            dot(8,'cyan')
            penup()
            forward(28)
    #the third column
    goto(x+24,y-8)
    for i in range(avg*4,line_len):
        if data[i]==0:
            dot(8,'pink')
            penup()
            forward(12)
        elif data[i]==1:
            dot(8,'cyan')
            penup()
            forward(12)
    hideturtle()
    return data[line_len:]
```

```
def draw_all(x,y,data):
    speed(0)
    penup()
    remain_data=draw_minus_sign(x,y-34,data,4)
    remain_data = draw_l(x + 70, y, remain_data, 6)
    remain_data = draw_o(x + 86, y-42, remain_data, 8)
    remain_data = draw_g(x + 136, y - 38, remain_data, 12)
    remain_data = draw_p(x + 190, y, remain_data, 10)
    done()

def main():
    screensize(600, 800)
    pensize(8)
    src="hello"   #Here put in “world” when encoding “world”.
    data=[int(i) for i in ''.join([format(ord(i),'08b') for i in src])]
    print("Binary sequence of original information：{}".format(data))
    assert len(data)==40,"The encoded information is five bytes"
  draw_all(-100,0,data)
```

The code for decoding "hello" and "world".

```
import cv2
import numpy as np

def ide_color(img_name):
    """
    function:Identify different color areas in the image
    param:img_name
    return: Recongnized different colors
    """
    img= cv2.imread(img_name)
    hsv = cv2.cvtColor(img, cv2.COLOR_BGR2HSV)
    # Set the thresholds of pink and cyan in the HSV color space respectively
    lower_pink=np.array([140,43,46])
    upper_pink=np.array([175,255,255])
    lower_cyan = np.array([80, 43, 46])
    upper_cyan = np.array([115, 255, 255])
    #Get the coordinates of different color areas in the picture
    dst_pink = cv2.inRange(src=hsv, lowerb=lower_pink, upperb=upper_pink)
    dst_cyan = cv2.inRange(src=hsv, lowerb=lower_cyan, upperb=upper_cyan)
    # cv2.imwrite('test/dst_red.jpg',dst_pink)
    # cv2.imwrite('test/dst_green.jpg',dst_cyan)
    cv2.imshow('dst_pink', dst_pink)
    cv2.imshow('dst_cyan', dst_cyan)
    cv2.waitKey(0)
    # cv2.destroyAllWindows()
    return dst_pink,dst_cyan

def ide_position(img):
    """
    function:Locate the coordinates of different color areas
    param:img_name
    return: The center coordinates and radius of a circle
    """
    circles = cv2.HoughCircles(img, cv2.HOUGH_GRADIENT, 1,10, param1=20, param2=6,
minRadius=5, maxRadius=15)
    circles = np.uint16(np.around(circles))
    #Mark the recognized color area with a circle
    for i in circles[0, :]:
        cv2.circle(img, (i[0], i[1]), i[2], (0, 255, 0), 1)
        cv2.circle(img, (i[0], i[1]), 1, (0, 0, 255), 1)
    cv2.imshow("circles", img)
    cv2.waitKey(0)
    return circles[0,:]

def cor_sort(lst,threshold):
    """
    function:The sorting of coding information sequence is realized by bubble sorting
algorithm.
    :param lst:
```

```
    :param:threshold
    :return: The binary sequence of decoded information
    """
    for i in range(1,len(lst)):
        for j in range(0,len(lst)-i-1):
            if abs(int(lst[j][0])-int(lst[j+1][0]))<=threshold:
                if lst[j][1]>lst[j+1][1]:
                    lst[j],lst[j+1]=lst[j+1],lst[j]
            else:
                if lst[j][0]>lst[j+1][0]:
                    lst[j],lst[j+1]=lst[j+1],lst[j]
    return lst

def decode(img_name):
    dst_pink, dst_cyan = ide_color(img_name)
    circles_pink=ide_position(dst_pink)
    #Organize the coordinates of the center of the pink area in the image and the corresponding
colors into a list.
    x_y_color_pink=[]
    for circle in circles_pink:
        x_y_color_pink.append((circle[0],circle[1],0))
    circles_cyan=ide_position(dst_cyan)
    #Organize the coordinates of the center of the cyan area in the image and the corresponding
colors into a list
    x_y_color_cyan=[]
    for circle in circles_cyan:
        x_y_color_cyan.append((circle[0],circle[1],1))
    x_y_color=x_y_color_pink+x_y_color_cyan
    bin_list=[]
    x_y_color_sorted=sorted(x_y_color,key=lambda x:(x[0],x[1]))
    print("y_x_color_sorted_1:{}.".format(x_y_color_sorted))
    x_y_color_sorted=cor_sort(x_y_color_sorted,7)
    print("y_x_color_sorted_2:{}".format(x_y_color_sorted))
    for i in x_y_color_sorted:
        bin_list.append(i[2])
    return bin_list

def main():
    img_name = "test/fig_19.jpg"
    bin_list = decode(img_name)
    print(bin_list)
    print(len(bin_list))
    bin_str=''.join([str(i) for  i in bin_list])
    src_byte=[int(bin_str[i:i+8],2) for i in range(0,len(bin_str),8)]
    print(src_byte)
    src=''.join([chr(i) for i in src_byte])
    print(src)

if __name__=="__main__":
    main()
```

The code for encoding the QR code of “Eu”.

```
import qrcode
def qr_code(data,img_path):
    qr = qrcode.QRCode(version=1,
                       error_correction=qrcode.constants.ERROR_CORRECT_H,
                       box_size=4,
                       border=4)

    qr.add_data(data)
    qr.make(fit=True)
    img = qr.make_image(fill_color='red', back_color='green')
    img.save(img_path)
    img.show()

if __name__=="__main__":
    data= 'Eu'
    img_path = 'qr_pub.png'
    qr_code(data,img_path)
```

The code for encoding the barcode.

```
Def encode(src,map_dic):
   #first part
   fluorescent=[]
   src_fir=src[:len(src)//2]
   encoder = Code128Encoder(src_fir)
   encoder.save("barcode.png", bar_width=6)

   src_sec=src[len(src)//2:]
   # second part
   src_sec_bin = [int(i) for I in ''.join([format(c, '08b') for c in src_sec.encode()])]
   print(src_sec_bin)
   for I in src_sec_bin:
      for key, value in map_dic.items():
         if I == key:
            fluorescent.append(value)
   print(fluorescent)

def main():
   src = "ciac"
   map_dic = {0: "red", 1: "green"}
   encode(src,map_dic)

if __name__=="__main__":
   main()
```

The code for decoding the barcode.

```
import cv2
from pyzbar import pyzbar
import numpy as np
from PIL import Image,ImageDraw,ImageFont
from os.path import splitext

def identify_barcode (img,decoded_img):
    """
    function: Identify barcodes in images
    :param :img,decode_img
    :return:Decoded information
    """
    gray_img=cv2.cvtColor(img,cv2.COLOR_BGR2GRAY)
    barcodes=pyzbar.decode(gray_img)
    barcode=barcodes[0]
    x,y,w,h=barcode.rect
    # cv2.rectangle(img, (x, y), (x + w, y + h), (255, 140, 0), 2)
    barcode_data=barcode.data.decode("utf-8")
    barcode_type=barcode.type
    img_PIL = Image.fromarray(cv2.cvtColor(img, cv2.COLOR_BGR2RGB))
    draw = ImageDraw.Draw(img_PIL)
    draw.text((x, y - 5), barcode_data, font=ImageFont.truetype('ARLRDBD.TTF', 16), 
fill=(255, 0, 0))
    img_PIL.save(decoded_img, 'jpeg')
    img = cv2.cvtColor(np.asarray(img_PIL), cv2.COLOR_RGB2BGR)
    cv2.imshow(str(decoded_img),img)
    cv2.waitKey(0)
    cv2.destroyAllWindows()
    print("type：{},include：{}".format(barcode_type, barcode_data))
    return barcode_data

def fluorescent_decode(img):
    color_range = {'red':{'Lower': np.array([120,60,80]), 'Upper': np.array([180,255,255])},
              'green':{'Lower': np.array([20, 20, 10]), 'Upper': np.array([100, 255, 255])},
              'black':{'Lower':np.array([0, 0, 0]),'Upper':np.array([180, 255, 46])},
              'white':{'Lower':np.array([0, 0, 221]),'Upper':np.array([180, 30, 255])}}
    if img is not None:
        gaussian_img=cv2.GaussianBlur(img,(5,5),0)
        hsv_img=cv2.cvtColor(gaussian_img,cv2.COLOR_BGR2HSV)

gray_img_red=cv2.inRange(hsv_img,color_range['red']['Lower'],color_range['red']['Upper'])
        gray_img_green = cv2.inRange(hsv_img, color_range['green']['Lower'], 
color_range['green']['Upper'])

        ret_red, binary_red = cv2.threshold(gray_img_red, 127, 255, cv2.THRESH_BINARY)
        kernel_red= cv2.getStructuringElement(cv2.MORPH_RECT, (10, 25))
        gradient_red = cv2.morphologyEx(binary_red, cv2.MORPH_GRADIENT, kernel_red)
        ret_green, binary_green = cv2.threshold(gray_img_green, 127, 255, 
cv2.THRESH_BINARY)
```

```
        kernel_green = cv2.getStructuringElement(cv2.MORPH_RECT, (10, 25))
        gradient_green = cv2.morphologyEx(binary_green, cv2.MORPH_GRADIENT,
kernel_green)

        contours_red, hierarchy = cv2.findContours(gradient_red, cv2.RETR_EXTERNAL,
cv2.CHAIN_APPROX_SIMPLE)
        contours_green, hierarchy = cv2.findContours(gradient_green, cv2.RETR_EXTERNAL,
cv2.CHAIN_APPROX_SIMPLE)
        fluorescent_list=[]
        for red in contours_red:
            x_red, y_red, w_red, h_red = cv2.boundingRect(red)
            cv2.rectangle(gray_img_red, (x_red, y_red), ((x_red + w_red), (y_red + h_red)), (100,
255, 255), 1)
            fluorescent_list.append((x_red, y_red, w_red, h_red,0))

        for green in contours_green:
            x_green, y_green, w_green, h_green = cv2.boundingRect(green)
            cv2.rectangle(gray_img_green, (x_green, y_green), ((x_green + w_green), (y_green +
h_green)), (100, 255, 255), 1)
            fluorescent_list.append((x_green, y_green, w_green, h_green,1))

        fluorescent_list=sorted(fluorescent_list, key=lambda x: x[0])
        print(fluorescent_list)
        print(len(fluorescent_list))
        cv2.imshow('gray_img_red',gray_img_red)
        cv2.imshow('gray_img_green',gray_img_green)
        cv2.waitKey(0)

        decoded_data_bin=''.join([str(x[-1]) for x in fluorescent_list])
        flu_decoded_bytes=[''.join(decoded_data_bin)[i:i+8] for i in
range(0,len(decoded_data_bin),8)]
        flu_decoded_data=''.join([chr(int(x,2)) for x in flu_decoded_bytes])
        print("fluorescence result ： {}".format(flu_decoded_data))
        return flu_decoded_data

def main():
    figure_name="figure2.jpg"
    decoded_figure=splitext(figure_name)[0] + "_decoded"+splitext(figure_name)[1]
    coded_img=cv2.imread(figure_name)
    barcode_res=identify_barcode(coded_img,decoded_figure)
    flu_decode_res=fluorescent_decode(coded_img)
    barcode_data=barcode_res+flu_decode_res
    print("finial result:{}".format(barcode_data))

if __name__=="__main__":
    main()
```

4. References

[1] J. Husson, J. Dehaudt, L. Guyard, *Nature Protocols* **2014**, *9*, 21.
[2] Z. Li, Z. Hou, H. Fan, H. Li, *Advanced Functional Materials* **2017**, *27*, 1604379.
